# Effectiveness of a continuum of care in maternal health services on the reduction of maternal and neonatal mortality: Systematic review and meta-analysis

**DOI:** 10.1016/j.heliyon.2023.e17559

**Published:** 2023-06-22

**Authors:** Muluwas Amentie Zelka, Alemayehu Work Yalew, Gurmesa Tura Debelew

**Affiliations:** a Department of Public Health, College of Health Sciences, Assosa University, Assosa, Ethiopia; b Department of Reproductive Health, School of Public Health, College of Health Sciences, Addis Ababa University, Addis Ababa, Ethiopia; c Department of Biostatistics and Epidemiology, School of Public Health, College of Health Sciences, Addis Ababa University, Addis Ababa, Ethiopia; d Department of Population and Family Health, Institute of Health, Jimma University, Jimma, Ethiopia

**Keywords:** Continuum, Health, Maternal, Mortality, Neonatal

## Abstract

**Background:**

Sustainable Development Goals −3 (SDG – 3) were to ensure healthy live and promote well-being by reducing global maternal and neonatal deaths. These were to be implemented through the concept of continuum of care in maternal health program framework to improve health outcomes. There is a paucity of published evidences; as such, this review is designed to assess the effectiveness of the concept of continuum of care in maternal and neonatal health services on the reduction of maternal and neonatal mortality.

**Methods:**

A search was conducted using the key words; maternal and neonatal, health services, continuum of care, maternal and neonatal mortality. Search focused on PubMed, Cochrane, MEDLINE and Google Scholar. Extractions of articles were done based on predetermine criteria. Data were compiled, and screened, entered and analysis was done using STATA 13 and Rev. Man. software. Effects of the intervention package were determined and the result was interpreted in random effect RR with 95%CI. The publication bias was determined by using funnel plot, Egger and Bagger test, heterogeneity, and sensitivity test.

**Results:**

A total of 4685 articles were retrieved of these 20 articles reviewed. Articles on 631,975 live births (LBs) were analyzed. Results showed the distribution as follows; 23,126 newborns died within 28 days resulting [NMR = 35/1000LBs among the intervention group whereas NMR = 39/1000LBs among the control group]. The pooled effect of the intervention was significantly reduced neonatal mortality (RR = 0.84; 95%CI: 0.77–0.91). Similarly, 1268 women died during the pregnancy period up to 42 days after childbirth that resulted [MMR = 330/100,000LBs among the intervention group whereas MMR = 460/100,000LBs among the control group]. The pooled effect of the intervention was not a statistically significant association with maternal mortality (RR = 0.64; 95%CI: 0.41–1.00).

**Conclusion:**

Adoption of continuum of care concepts in maternal health services reduced maternal and neonatal mortality. We recommend strengthening and effective implementation of a continuum of care in maternal health services to improve maternal and neonatal health care outcomes.

## 1 Introduction

The Sustainable Development Goal (SDG – 3) will ensure healthy live and promote well-being of human being [1]. This goal calls for achieving universal access to sexual and reproductive health care in order to reduce global maternal deaths, neonatal deaths, and reproductive health problems in developing countries. Besides, the goal target at the end of 2030, reduce the global maternal mortality ratio to less than 70 per 100,000 live births (LBs). Hence, the health and well-being of women matter to every person, society, and country [1].

Globally, the maternal mortality ratio (MMR) was reduced nearly by 44% from 385 maternal deaths per 100,000LBs in 1990 to 216 maternal deaths per 100,000LBs in 2015 [2]. Annually, millions of women and newborns die from preventable causes that can manage by easily and affordable interventions. Those interventions can save their lives, but it cannot be available for the target group as they need. Around 4 million of babies die within the first month of life and more than 3 million at birth encountered as stillbirth. Thus, 99% of maternal, newborn, and child deaths occur in developing countries [3,4].

Estimated maternal mortality indicated that there has been a significant variability within the region, which reflects inequities in access to health services. In high-income countries, the completion rate of a continuum of care in maternal health services is the highest in the world: almost all women attend at least 4th ANC visit and receive services by skilled providers during childbirth and postpartum, whereas in low-income countries, the completion rate of a continuum of care in maternal health services is very low in the world: only 40% of all pregnant women receive the recommended antenatal care visits, of them few women return for skilled care during childbirth and PNC services [5]**.** As a result, every day, 800 women die from causes related to pregnancy and childbirth. However, in Sub-Saharan Africa, approximately 550 women die every day from preventable causes, which accompanies 66% of worldwide maternal deaths. Children under the age of five in Sub-Saharan Africa are 16 times more likely to die than in high-income countries due to poor access to quality of services and discontinuation of maternal health services [3].

In spite of global attention and priorities for maternal and child health programs, still the reduction of maternal and neonatal mortality is very slow in developing countries including Ethiopia [6]. Even though, the progresses of maternal and neonatal health are minimal across countries; continuum of care is a key strategy in maternal and child health programs. Thus, it provides a framework to improve maternal and neonatal health outcomes [7]. Even though, neonatal health is heavily intertwined with maternal health; global efforts are moving toward the implementation of package approaches along with the continuum of care from pre-pregnancy to childhood period is a crucial strategies [8]. Hence, a continuum of care is a new paradigm shift designed in maternal, neonatal, and child health programs to avert or minimize the big challenges encountered in this program [4,7]. Thus, an effective provision of a continuum of care in maternal health services for women and newborns at the health facility can avert an estimate of 113,000 maternal deaths, 531,000 stillbirths, and 1.325 million neonatal deaths annually by 2020 [9].

Hence, the continuum of care approach can prevent millions of needless deaths and disabilities. This approach promotes care for mothers and their children from pregnancy to delivery and postnatal period, which is an essential step towards the promotion of childhood health and a productive life [4,7,10]. In another direction, the continuum of care approach is an intervention that links households to hospital by improving home-based practices, mobilizing families to seek care as they need, and increasing access to and quality of care at health facilities; and between the place of care given (households and community, outpatient and outreach services, and clinical care setting) [4,7,8,11]. Thus, the continuum of care has two dimensions: the first one is the time dimension: continuity of care over time for women, newborns, and children; and the second one is the space dimension: integrated service delivery provided by health facilities and communities [4,7,8,10].

During pregnancy, all pregnant women should receive ANC services from skilled providers, given their birth at a health facility, and attended by skilled providers who manage normal delivery and to refer for complication cases and continues receiving care after delivery for themselves and their newborn within the postnatal period, the critical time for reducing neonatal and maternal mortality [10,11]. Therefore, a continuum of care is one of the key programmatic strategies for improving maternal and neonatal health outcomes [10,11].

In-spite of maternal and neonatal health is a good indicator for population health status, still maternal and neonatal mortality are the highest in the worldwide. During the era of Sustainable Development Goals (SDGs), assessing the effectiveness of a continuum of care in maternal health services on maternal and neonatal health outcomes is very crucial for policy makers to design evidence-based health interventions for their country.

The specific aim of this review is to determine the effectiveness of continuum of care in maternal health services on the reduction of maternal and neonatal mortality. Hence, the review questions, which are answered at the end of this review: Is completion of a continuum of care in maternal health services effective in reducing maternal mortality? Is completion of a continuity of maternal health services effective in the reduction of neonatal mortality?

## 2 Methodology

### 2.1 Selection and study entry criteria

In this systematic review and meta-analysis, the authors strictly adhered to PICO approach questions to declare the inclusion and exclusion criteria. Hence, the eligibility criteria must address the question of study type, participants, interventions, comparison/controls and outcomes (PICO) [12]. Similarly, to ensure the improvement of the reported systematic reviews and meta – analysis, PRISMA guideline was used, particularly it is useful to evaluate the interventions and critical appraisal of published reviews [13].

#### 2.1.1 Type of studies

The type of studies included in the review are mainly case – controls, follow-up studies, cohort studies, RCT studies, and interventional studies. In addition, studies that provided an estimation of maternal and neonatal mortality levels derived from direct counting or from special surveys that linked to the continuum of care in maternal health services are considered for inclusion.

#### 2.1.2 Type of participants

The study participants were women in childbearing age who gave at least one birth experience and neonates.

#### 2.1.3 Type of interventions and controls

The type of intervention targeted in this study were pregnant women who were continuously using maternal health services (ANC, institutional delivery service, and PNC) throughout the pregnant stage up to the postnatal period and offering those packages of interventions at the health facility, home and community level considered as an intervention group whereas women who were not using any component of maternal health services or discontinuity of maternal health services considered as the control group. Moreover, different health interventions/strategies that used to improve maternal and child health program and increase the completion rate of maternal health services/packages were considered as the intervention group (Table 1).

**Table 1:** Description of interventions type for each study included in systematic review and meta-analysis on neonatal mortality, 2004–2018.

S. No	Authors/Years of Publication	Completion of Continuum of care in maternal health services considered as intervention group type
		Type of interventions considered
1	Tripathy, P. et al., 2010	Provision of training for women group facilitators who assisted or support women groups to identify and prioritize maternal and neonatal problems. Then, helped women’s groups to identify possible solutions and to plan, implement and monitor solution strategies for maternal health services in the community which improve completion rate of continuity of maternal health services
2	Soofi, S. et al., 2017	The interventions offered for women: the lady health workers (LHWs) support and train on recognition of high-risk pregnancies and neonatal danger; promotion of antenatal care and use of iron folic acid during pregnancy; promotion of adequate maternal diet and rest; provision of clean delivery kit to pregnant women; Immediate neonatal care; promotion of exclusive and early breastfeeding; cord care (dry, clean, and avoid any traditional application); delayed bathing; LHWs present at home births; Domiciliary care with bag and mask for asphyxiated neonates and referral for aftercare; improved thermal care; provision of first dose of amoxicillin to suspected infected neonates and referral to referral hospital; daily follow-up and provision of amoxicillin for 7 days in case of refused referral to hospital; provision of inflatable bag and mask, sucker bulb, amoxicillin, clean delivery kits, and management protocols to LHWs; support group (health education) training Exclusive training on support group methods, communication, and counseling skills for LHWs; male motivators training; orientation for traditional birth attendants (dais) Basic essential neonatal care training and linkage with LHWs; Health facility strengthening Health-care providers training on essential neonatal care and management of birth asphyxia, low-birth weight babies, and neonatal sepsis.
3	Persson, L.A. et al., 2013	Community dialogues and discussion was made monthly to improve maternal health care practice. Community health care staffs that were responsible and countable for health community health and key person in the community are act as facilitators to make efforts to improve utilization of maternal health services. A problem-solving approach was employed.
4	Manandhar,D.S. et al., 2004	Activate a women’s group through an action learning cycle and conducting monthly women’s group meetings with female facilitators who assisted the women’s group in identifying and prioritizing maternity and neonatal health issues. An also assisting the women’s group in identifying potential solutions as well as planning, implementing, and monitoring community-based solutions by government health workers, female community health volunteers, and traditional birth attendants. The overall intervention targeted to improve maternal health services and scale up utilization rate
5	Kumar, V. et al., 2008	Interventions offered for intervention group: provision of an essential maternal and newborn care packages (birth preparedness, clean delivery and cord care, thermal care, breastfeeding promotion, danger sign recognition); distribution of a liquid crystal hypothermia indicator (ThermoSpot) by community health workers through collective meeting; community meetings, folk song meetings, community volunteer meetings; two ANC and two PNC home visits
6	Kikuchi, K. et al., 2016	Interventions comprised continuum of care in maternal health services via time and space dimensions. Hence, maternal health intervention packages/services that addressed women’s time dimension from pre-pregnancy to pregnancy and also he space dimension of continuum of care comprised three care stages—community/family care, outpatient/outreach care, and clinic care. The community/family care interventions addressing pre-pregnant women’s nutrition, health education for family planning and reproductive health, and prevention of HIV/STIs at home or community level. The outpatient/outreach care comprised family planning and prevention/management of HIV/STIs. The clinical care interventions included elective abortion and post-abortion care through facility-based care at primary and referral levels
7	Kikuchi, K. et al., 2015	Packages of maternal health services such as ANC, skilled delivery and PNC services were offered for women and those who were received the whole maternal health service via time dimension considered for interventions. And also, maternal health services provision via different modality starting from home visit up to specialized hospital and the linkage of those health facility considered as interventions
8	Jokhio, A.H. et al., 2005	Provision of training for trained birth attendants on ANC, delivery care, PNC; clean delivery; use of disposable delivery kits; refer women for emergency obstetrical care; care of the newborn. And also ask women to visit at least three times during pregnancy; linkage of TBA with lady health workers and make ready outreach clinics for ANC.
9	Bhutta, Z.A. et al., 2011	Female health workers provided group health education sessions for women on antenatal care and maternal health. Provision of clean delivery kits, promotion of health facility delivery, immediate newborn care, and training in the identification of danger signs; instruction on antenatal and postnatal home visits for female health workers; training for traditional birth attendants on basic newborn care and also establishing community health committees for maternal and newborn care.
10	Baqui, A.H. et al., 2008	During home visit, health workers promote at least 3 ANC for mothers/families; encourage families to call auxiliary nurse-midwife or trained TBAs to attend delivery; home visit PNC after birth during 0–27 days; follow-up visits for sick.
11	Baqui, A.H. et al., 2008	Providing training for community health workers; applying home based care model service delivery group such as provide ANC home visit, PNC visit, referral of sick newborns to HF and treatment in the house with injectable antibiotics. Moreover, applying community based care model service delivery group such as TBA orientation on cleanliness during delivery, danger sign, newborn care, community meeting with pregnant women by community mobilisers.
12	Azad, K. et al., 2010	Women’s groups provided maternal and neonatal health promotion for women in reproductive age group at the community level. Providing training for traditional birth attendants on safe deliveries and resuscitation of newborns with symptoms of birth asphyxia using the bag valve mask. And also, basic and refresher clinical training for health workers on essential components of maternal and neonatal health care.
13	Kirkowood, B.R. et al., 2013	Provision of training CBSVs; identify pregnant women in the community; two home visits during pregnancy; three PNC home visits on days 1, 3 and 7; developing a sustainable supervisory and remuneration structure for the CBSVs; sensitization of HF staff to intervention messages and approach; sensitization of TBAs.
14	Colbourn, T. et al., 2013	Established participatory women’s groups to mobilize communities around maternal and newborn health services, using 81 volunteer facilitators, supported by nine staff, across the allocated clusters. The facilitators each formed nine village women’s groups which followed an “*action cycle*” to identify and prioritize maternal and neonatal health problems, decide upon local solutions, advocate for, implement and evaluate such strategies. The overall strategies/interventions were used to improve maternal and child health services and completion rate of continuum of care via time and space dimension
15	Lewycka, S. et al., 2013	Providing training for women’s group to identify and priorities MCH problem, identify strategies to implement, plan and implement them accordingly; training volunteer peer counselors made home visits 5 times during early pregnancy, 1 week, 1, 3, 5 months after birth for health education such as exclusive breast feeding, infant care, immunizations, PMTCT and family planning. And also, health surveillance assistants of district health office conduct regular supervision of counselors.
16	Kumar, V. et al., 2012	Birth preparedness and make ready emergency preparedness (identifying health facility, money, transport), obtaining a clean delivery kit, clothes and mattress for mother and newborn. Clean and adequate clothing available to mother & newborn as it is meant to be discarded after the confinement period. Providing maternal health care during postpartum period, including practice of early recognition of danger sign and care-seeking. Home visits on the day of delivery and day 3 were conducted to support promoted practices, and check the condition of the newborn and mother. The family was advised to seek care from health facility. Facilitated community meetings provided opportunities for pregnant women and their families to interact with each other and with health system workers and traditional care providers, to discuss danger signs, to share experiences and concerns, and to devise common strategies.
17	Eriksson, L. et al., 2018	The Neonatal Knowledge Into Practice (Neo-KIP) intervention was integrated into the existing health system and built on local community engagement for maternal and newborn health services/package. The facilitators groups mobilized and supported the groups in applying the Plan Do-Study-Act method, which included identifying and prioritizing problems (Plan), undertaking planned actions (Do) and finally evaluating the effect and reconsidering the problems and actions (Study and Act). This intervention was designed to enable integration into the health system to increase the possibilities of having sustained effects on maternal and child health services through time and space dimension of continuum of care.
18	Kabo, L. et al., 2016	The interventions to improve maternal and child health services: (i) implement SBM-R set performance standards based on annual assessment data, (ii) the use of maternal and newborn health (MNH) service delivery practices based on data from health facility registers and supportive supervision and (iii) MNH outcomes based on routine service statistics.
19	Patel, A.B. et al., 2017	Implementing continuum of care in maternal health services via space dimensions such that Inter- institutional referral was made to transfer a woman from a first or second level facility where she was admitted for labor and delivery to facility providing higher level of care, after admission to the day of delivery.
20	Fottrell, E. et al., 2013	Women’s groups at a coverage of 1 per 309 population that proceed through a participatory learning and action cycle in which they prioritize issues that affected maternal and neonatal health and design and implement strategies to address these issues. The nature of the intervention means that allocation was not masked. All unions, irrespective of allocation arm, received health system– strengthening initiatives from perinatal care project. These included the provision of basic medical equipment, traditional birth attendant training in essential newborn care, physician training, and establishing links between communities and health services.

#### 2.1.4 Type of outcomes

Two types of outcomes considered in this review were ***maternal mortality***, which is explained by according to ICD 10 – MM [14] and the ***neonatal mortality***, which is either early or late neonatal mortality which described by according to ICD 10 – PM [15].

#### 2.1.5 Search strategies for identification of studies

The searching strategies were done by searching only published studies which were reported from 2000 to 2018 in the form of English language. The searching engines used for this review were PubMed, Cochrane, MEDLINE and Google Scholar. Particularly in maternal mortality and morbidity review: MEDLINE is searching beyond the major electronic databases to identify studies done in less developed countries [16]. We used the following searching terms combined with the relevant medical subject headings (MeSH) with additional text words were used: ‘*pregnant women’, ‘mother’, ‘neonate’, ‘newborn’, ‘community’, ‘continuum of care’, ‘maternal health’, ‘neonatal health’, ‘maternal mortality’, ‘maternal death’, ‘neonatal mortality’, ‘neonatal death’, ‘continuum of care in maternal health’, ‘effect of continuum of care’* and ‘*contribution of continuum of care’*. Furthermore, bibliographies of the eligible papers were manually identified from relevant citations that our search missed.

### 2.2 Exclusion criteria from the review

The studies are excluded if and only if any of the following preconditions were apply: data were collected before 2000G.C; the number of study participants were less than 1000 (this criteria was set because the outcome variables are rare events); the study design used in the form of cross-sectional, case series, case report and any magnitude of the problems stated without linking with exposure factors; methodology of the study was not clear and any qualitative study and the outcome variables were not clearly stated by linking the exposure factors. Moreover, studies with a low quality score on the Newcastle–Ottawa quality assessment scale (less than 7) were excluded.

### 2.3 Data collection

#### 2.3.1 Data screening and selection process of the studies

**Fig. 1:**
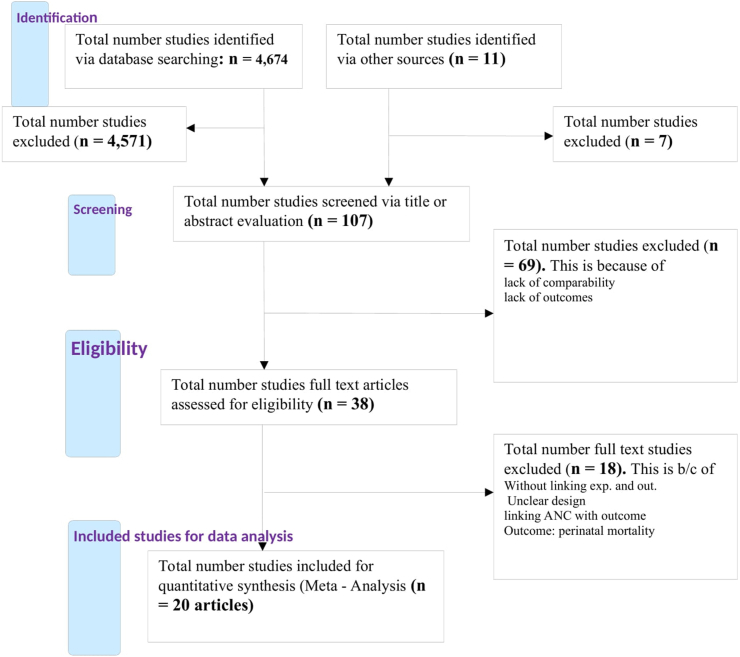
Flow chart of selection process of the studies for systematic review and meta-analysis (2000 – 2018).

The authors conducted the searching process, then after, evaluated all identified studies on the basis of titles and/or abstracts against the eligibility criteria. Those articles that fulfill the criteria of inclusion were included in the review but not fulfill the inclusion criteria were excluded from the review and mention the reason. The overall study selection process of the study was summarized in a flowchart (Fig. 1).

#### 2.3.2 Data extraction and management

The authors designed a data extraction tool for this review. The tool consisted of the name of the study authors, the year of publication, study areas, study designs, number of participants, number of events encountered within the exposure group, and non-exposure group. Hence, this tool makes it easy to manage, gather information, and facilitate data entry into the software (*supplementary-I*).

### 2.4 Assessment of risk of bias

Risk of bias was done using the “risk of bias” tool presented in the Cochrane Handbook for Systematic Reviews of Interventions [12]. For the included studies, quality appraisal was conducted by extracting information on study design, sampling method, source of data, and completeness of follow-up/record, reported definition, diagnostic procedures regarding outcome measurement, representativeness of exposed and non-exposed groups, ascertainment of exposure and outcomes/results. Hence, all studies considered in the systematic review and meta-analysis were quality scores greater than or equal to 7.

### 2.5 Measurement of treatment effect

Measurement of intervention effect on the outcomes was determined for all studies included and presented the result as a random effect risk ratio (RR) with 95% confidence intervals (CI).

### 2.6 Statistical methods and data analysis

Data were analyzed by using *Rev.Man. 5.3* software and *STATA Meta 14.1* software. First, descriptive data synthesis was done for each study. Secondly, by aggregating each study and analyzed the whole study by forest pilot analysis to produce the pool effect of the interventions on the outcomes. Hence, a random effect model was used to determine the pooled effect of an intervention on the outcome, which expressed as RR with 95%CI and at a significant level (p < 0.05). The publication bias was determined by using funnel plot, Egger’s and Bagger’s test. The Funnel plot is simply a graphical representation to detect the presence of publication bias [17]. Moreover, I^2^ test was used to test heterogeneity between the studied (*I*^*2*^ *= 87%, p < 0.0001*). Even though, the source of heterogeneity was assessed using subgroup analysis, but the possible source of heterogeneity was not detected. However, sensitivity analysis was performed to detect/identify the effect of individual studies on the overall pool effects of an intervention on the outcome.

### 2.7 Ethical consideration and approval

The ethical approval of this review was granted from Research Review of Ethical committee (REC) at School of Public Health level, as part of Institutional Review Board (IRB) of College of Health Science, Addis Ababa University, with the protocol number of SPH/3089/11.

## 3 Result

### 3.1 Description of the studies

A total of 4685 articles were retrieved from different sources of databases (Fig. 1). Among them, ***4,647*** of the studies were identified from PubMed; ***27*** articles were from other sources and ***11*** of the articles were from Google Scholar searching. After identification, 4578 of the articles were removed due to duplication of the articles and their titles, which found to be irrelevant for this review question. Among the remaining articles; 107 studies were assessed and screened based on the relevant value of the abstracts, but 69 articles were removed because the abstract is irrelevant on the base of exposure and outcome variables. Then, the remaining 38 articles were assessed for eligibility by reviewing the full text of the article; however, 18 articles were excluded because they did not fulfill the present criteria such that they didn’t have an intervention or treatment, no relevant design, no clear comparison group and had no clear outcome. Lastly, 20 articles were fulfilled the eligibility criteria and considered in the qualitative systematic review. Among the studies considered for the systematic review and meta-analysis, 12 of the studies linked continuum of care in maternal health services with maternal mortality which considered as the outcome variable, whereas 20 of the studies linked continuity of maternal health services with neonatal mortality which considered as the outcome variable.

Among the twenty studies, 19 articles were retrieved from low and middle income countries. Of them, four articles were from Africa countries: Ghana [18]; Malawi [19]; Nigeria [20] and Rural Malawi [21] and thirteen articles were from Asia countries: India [[22], [23], [24], [25]]; Rural Pakistan [26,27]; Nepal [28]; Pakistan [29]; Rural Northern India [30]; Bangladesh [[31], [32], [33]] and Vietnam [34]. And one articles from Europe, Sweden [35] and two articles were a systematic review and meta-analysis which conducted in low and middle income countries LMICs [36,37].

### 3.2 Neonatal mortality

In this review, a total 631,975 live births occurred among 757,877 study participants. In the intervention group, 318,773 of live births happened, of them 11,010 of the newborns died within 28weeks, having a NMR was 35 deaths per 1000 LBs. Similarly, 313,202 of live births occurred in the control group, of whom 12,116 of the newborns died within 28weeks, having a NMR being 39 deaths per 1,000LBs (Table 2).

**Table 2:** Characteristics of included articles for systematic review and meta-analysis on neonatal mortality, 2004–2018.

S.No	Authors/Years of Publication	Country	Design	Sample Size		Continuum of care		Discontinuity of care		Overall total		NMR
				Intervention	Control	Neonatal deaths	Live births	Neonatal deaths	Live births	Neonatal deaths	Live births	
1	Tripathy, 2010	India	Cluster RCT	9770	9260	406	9469	531	8980	937	18,449	50.8*
2	Soofi, 2017	Rural Pakistan	Cluster RCT	24,792	26,644	736	17,705	1050	19,163	1786	36,868	48.4*
3	Persson, 2013	Sweden	Cluster RCT	11,906	10,655	195	11,818	194	10,559	389	22,377	17.4*
4	Manandhar, 2004	Nepal	Cluster RCT	3036	3344	76	2899	119	3226	195	6125	31.8*
5	Kumar, 2008	India	Cluster RCT	2716	1143	112	2609	91	1079	203	3688	55.0*
6	Kikuchi, 2016	LMICs	Quasi and RCT	42,400	40,396	1624	42,400	1890	40,396	3514	82,796	42.4*
7	Kikuchi, 2015	LMICs	Quasi and RCT	109,322	100,704	3563	109,322	3891	100,704	7454	210,026	35.5*
8	Jokhio, 2005	Pakistan	Cluster RCT	10,114	9443	340	9710	439	8989	779	18,699	41.7*
9	Bhutta, 2011	Rural Pakistan	Cluster RCT	14,152	12,835	517	12,028	540	11,005	1057	23,033	45.9*
10	Baqui, 2008	Rural Northern India	Quasi RCT	13,826	14,952	393	7812	299	6014	692	13,826	50.1*
11	Baqui, 2008	Bangladesh	Cluster RCT	75,218	37,598	1368	31,094	696	15,350	2064	46,444	44.4*
12	Azad, 2010	Bangladesh	Cluster RCT	20,943	22,774	570	16,926	656	17,967	1226	34,893	35.1*
13	Kirkowood, 2013	Ghana	Cluster RCT	9174	9435	230	7898	252	7721	482	15,619	30.9*
14	Colbourn, 2013	Malawi	Cluster RCT	14,524	4192	138	5107	162	4766	300	9873	30.4*
15	Lewycka, 2013	Rural Malawi	Cluster RCT	5772	6310	125	4494	147	4960	272	9454	28.8*
16	Kumar, 2012	India	Cluster RCT	2749	1142	112	2609	91	1079	203	3688	55.0*
17	Eriksson, 2018	Vietnam	Follow up study	11,818	10,559	195	11,818	194	10,559	389	22,377	17.4*
18	Kabo, 2016	Nigeria	Follow up study	1000	1000	2	1000	9	1000	11	2000	5.5*
19	Patel, 2017	India	Cohort study	3236	31,083	121	3236	594	31,083	715	34,319	20.8*
20	Fottrell, 2013	Bangladesh	Cluster RCT	9106	8834	187	8819	271	8602	458	17,421	26.3*
				**395,574**	**362,303**	**11,010**	**318,773**	**12,116**	**313,202**	**23,126**	**631,975**	36.6*

**Note:** * per 1,000LB

**Abbreviations:** RCT, Randomized Clinical Trial; NMR, Neonatal Mortality Rate; LMICs, Low – Middle Income Countries; LB, Live Birth.

Besides, eleven studies revealed that completion of continuum of care in maternal health services had a statistically significant association with the reduction of neonatal mortality [19,[22], [23], [24],[26], [27], [28], [29],^31^,^36^,^37^]. Whereas, eight studies indicated that interventions were not showing any statistical significant association with the reduction of neonatal mortality [18,20,21,30,[32], [33], [34], [35]] but surprisingly one article revealed that the interventions resulted in a statistically significant increased occurrence of neonatal mortality as compared with discontinuation of maternal health services [25].

**Fig. 2:**
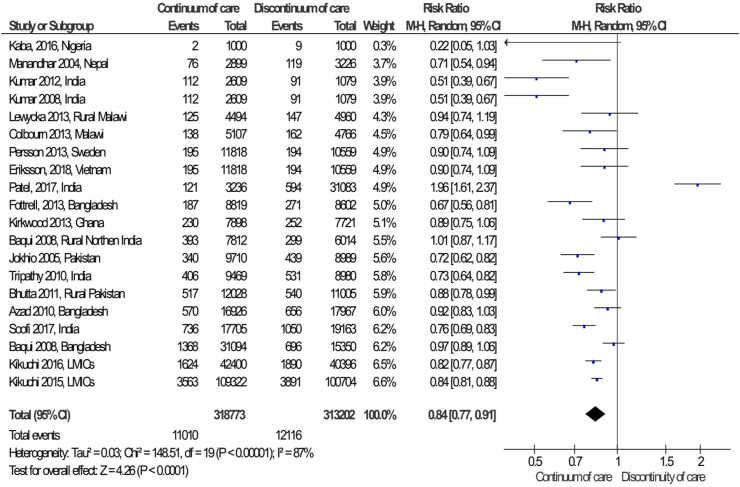
Forest Plot on random effect of continuity of maternal health services on neonatal mortality, 2000-2018.

**Fig. 3:**
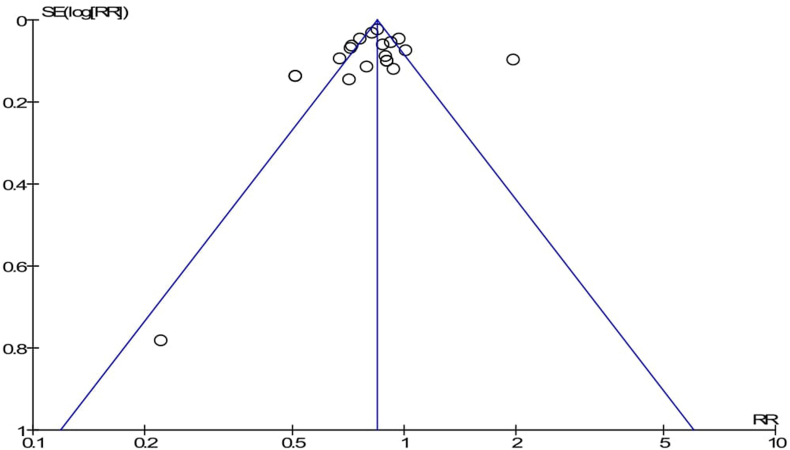
Funnel plot of meta-analysis on effect of continuity of maternal health services on neonatal mortality, 2000-2018.

**Fig. 4:**
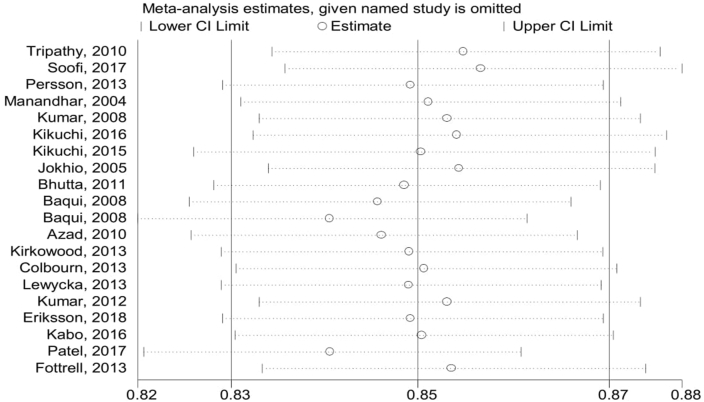
Sensitivity test for a single study effect on overall pooled effect.

The pooled effects of neonatal mortality among the intervention packages were a statistically significant association with the reduction of neonatal mortality compared to the control group (RR = 0.84; 95%CI: 0.77–0.91) (Fig. 2). Simply, the graphical observation on the funnel plot showed that symmetry on the pooled line, which is indicating that no publication bias (Fig. 3). Moreover, the Begger and Egger tests were not statistically significant association with *p-value = 0.163* and *p-value = 0.715,* respectively. This indicated that no publication bias. Furthermore, a sensitive test was conducted to identify the influence of each study in meta-analysis, but there is no influential study on the overall pooled effect (Fig. 4).

### 3.3 Maternal mortality

In this review, twelve studies were considered for the analysis of a maternal mortality outcome; a total of 317,742 live births were given by the study participants. From them, 1268 maternal deaths occurred within a pregnant period up to 42 days after childbirth that resulted in a maternal mortality ratio 399 deaths per 100,000LBs. Hence, the maternal mortality ratio among the intervention group (completion of continuum of care in maternal health services) was 330 deaths per 100,000LBs, whereas among the control group MMR was 460 deaths per 100,000LBs (Table 3).

**Table 3:** Characteristics of study articles for systematic review and meta-analysis on maternal mortality, 2004–2018.

S.No	Authors/Years of Publication	Country	Design	Sample Size		Continuum of care		Discontinuity of care		Over all Total		MMR
				Intervention	Control	Maternal deaths	Live births	Maternal deaths	Live births	Deaths	Live birth	
1	Tripathy, 2010	India	Cluster RCT	9770	9260	60	9469	109	8980	169	18,449	916**
2	Manandhar, 2004	Nepal	Cluster RCT	3036	3344	2	2899	11	3226	13	6125	212**
3	Kikuchi, 2016	LMICs	Quasi and RCT	27,738	27,051	106	27,738	103	27,051	209	54,789	381**
4	Kikuchi, 2015	LMICs	Quasi and RCT	54,756	50,280	145	54,756	153	50,280	298	105,036	284**
5	Jokhio, 2005	Pakistan	Cluster RCT	10,114	9443	27	9710	34	8989	61	18,699	326**
6	Azad, 2010	Bangladesh	Cluster RCT	20,943	22,774	55	15,153	32	14,736	87	29,889	291**
7	Colbourn 2013	Malawi	Cluster RCT	14,524	4192	11	5107	10	4766	21	9873	213**
8	Lewycka, 2013	Rural Malawi	Cluster RCT	5772	6310	23	4494	29	4960	52	9454	550**
9	Kumar, 2012	India	Cluster RCT	2749	1142	12	2609	10	1079	22	3688	597**
10	Kabo, 2016	Nigeria	Follow up study	5000	5000	35	5000	251	5000	286	10,000	2860**
11	Patel, 2017	India	Cohort study	3236	31,083	1	3236	12	31,083	13	34,319	38**
12	Fottrell, 2013	Bangladesh	Cluster RCT	9106	8834	14	8819	23	8602	37	17,421	212**
				**166,744**	**78,713**	**491**	**48,990**	**777**	**68,752**	**1268**	**317,742**	399**

**Note:** ** per 100,000LB

Abbreviations: RCT, Randomized Clinical Trial; NMR, Neonatal Mortality Rate; LMICs, Low – Middle Income Countries; LB, Live Birth.

Based on this review technique, three studies indicated that the intervention packages resulted in a statistically significant reduction on maternal mortality [20,24,28] whereas eight studies were not a statistically significant association with reduction of maternal mortality [19,21,22,25,29,31,36,37]. However, to your surprising, one study revealed that the intervention packages had been significantly increased maternal mortality as compared with the control group [33].

**Fig. 5:**
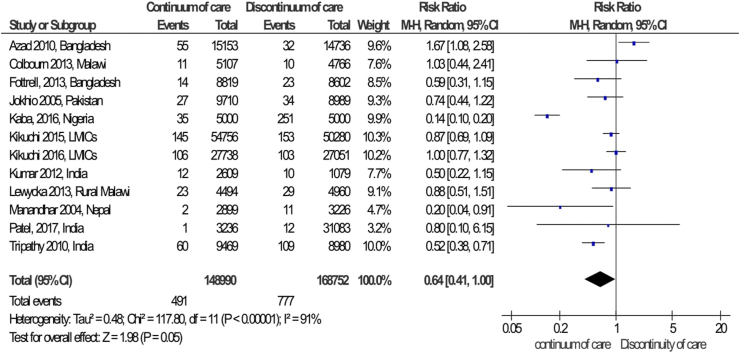
Forest Plot on random effect of continuity of maternal health services on maternal mortality, 2000-2018.

**Fig. 6:**
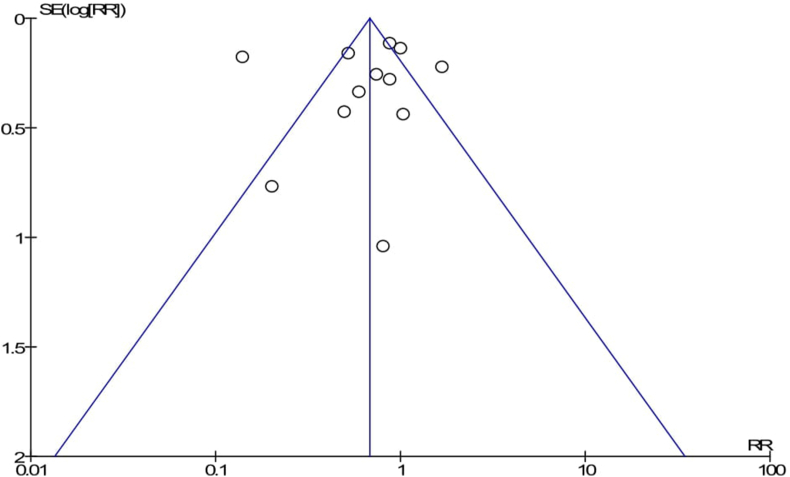
Funnel plot of meta-analysis on effect of continuity of maternal health services on maternal mortality, 2004-2018.

**Fig. 7:**
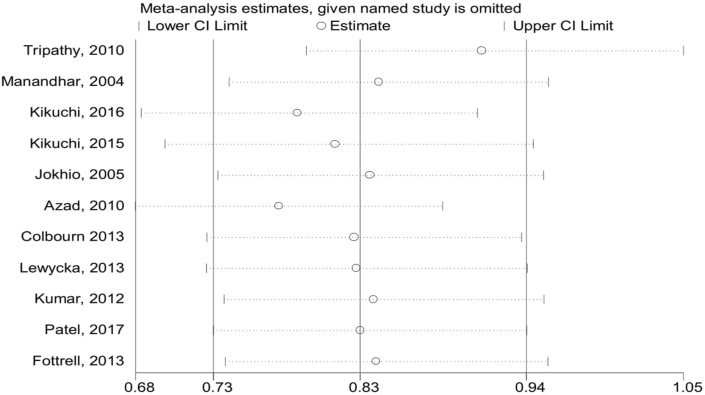
Sensitivity test for a single study effect on overall pooled effect.

The pooled effects of completion of a continuum of care in maternal health services were not a statistically significant association with the reduction of maternal mortality as compared to discontinuation of care in maternal health services (RR = 0.64; 95%CI: 0.41–1.00) (Fig. 5). However, completion of a continuum of care in maternal health services was a clinically significant association with the reduction of maternal mortality and neonatal mortality. The publication bias was detected by using a funnel plot, it looks like symmetrically distributed on both sides of the combined effect size line, which indicated that no publication bias (Fig. 6). Furthermore, the Begger and Egger tests were not statistically significant with *p-value = 0.451* and *p-value = 0.758,* respectively. This also revealed that no publication bias. In addition, a sensitive test was done to detect the influence of the individual studies on the overall pooled effect. Thus, there is no individual influential study (Fig. 7).

## 4 Discussion

This systematic review and meta-analysis revealed that the intervention packages were significant association with reduction of neonatal mortality compared to the controlled group.

In this review, the magnitude of neonatal mortality among the intervention group NMR = 35 deaths per 1,000LBs, whereas among the controlled group NMR = 39 deaths per 1000LBs. This evidence is consistent with the specific studies conducted in Southwest Ethiopia (NMR = 35.5 deaths per 1000LBs) [38] and Benishangul Gumuz region (NMR = 35 deaths per 1000LBs) [39]. However, this finding is higher than NMR reported by EDHS 2016 (29 deaths per 1000LBs) [39], in India state of Bihar (32.2 deaths per 1000LBs) [40] and extremely higher than a previous review conducted in low-middle income countries (NMR = 11.32 deaths per 1,000LBs) [41]. This variation is due to in this review we included only the longitudinal study design which is more accurate than cross-sectional study design. However, in the previous review they included a cross-sectional study design: almost half the articles (9/19) were cross-sectional study designs. Because of this, the review results did not comparable.

The findings of this review demonstrated that the intervention (completion of a continuum of care in maternal health services) had been a statistically significant effect on the reduction of neonatal mortality, which revealed that 16% reduction in the risk of neonatal mortality. However, this finding is consistent with evidence that documented in developing and low-middle income countries (71% of neonatal deaths were averted per year) [9]. Moreover, cost-effective intervention packages of maternal health services may be able to prevent up to 75% of neonatal deaths [42]. Similarly, provision of maternal health services via community-based approaches during pregnancy, during and after child birth has been addressed the burden of maternal, neonatal, and child morbidity and mortality in low and middle income countries [43]. Moreover, the package of maternal and child health interventions offered for pregnant women and after child birth for both women and babies should reduce neonatal mortality by 54% [23].

Different evidences supported that the continuum of care in maternal health services has a positive impact on promoting maternal and neonatal health [44]. Thus, it is a core aspect used to assess the progress of health goals: reduction of maternal and neonatal mortality. Likewise, continuum of care is a life-saving intervention package that targets against major causes of maternal and newborn mortality and morbidity [[45], [46], [47]]. In Japan, the maternal and neonatal mortality rate is extremely low, which suggests that the coverage of the continuum of care in maternal health services is very high. This indicates that the provision of a continuum of care in maternal health services has been a negative association with maternal and neonatal mortality [48,49]. In turn, women and newborns are highly vulnerable during child birth and immediately after childbirth, so the provision of a continuum of care at these times is very crucial and eliminates pregnant related problems and infectious diseases. Hence, it provides a good opportunity for the newborn to grow in the healthy life and reduce neonatal death and illness [50].

Previous systematic review and meta–analysis evidence in India document that integrating community-based neonatal interventions and facility-based interventions would reduce neonatal mortality. Hence, the efficacy of the intervention packages showed a significant reduction of neonatal mortality by 15% [51]. However, other evidence indicates that the combined effect size of facility delivery has a statistically significant association with the reduction of neonatal mortality by 26% as compared with home delivery [41]. This is because of the effective intervention of the maternal health service packages that would reduce neonatal mortality and morbidity by managing childbirth problems and treating infectious and malnutrition. Even though delivering these essential intervention packages by skilled providers at different levels (home, community, and health facilities), every target peoples are addressed and receive the service as they need. Thus, neonatal mortality rate can be averted [6].

Moreover, home-to-hospital continuum of care showed that an effective strategy in deciding appropriate health care accessible to women and newborns across the time and space dimension to improve maternal and neonatal health outcomes [52]. Hence, by integrating those health interventions and harmonizing the intervention setting can be more effective in the reduction of neonatal morbidity and mortality. Thus, the overall effect of the intervention packages can reduce neonatal mortality by 12% [53].

Similarly in Ethiopia, as a result of low coverage of high impact maternal and child health interventions, annually, 15,000 maternal deaths and 83,000 newborn deaths were happened. In turn, improving the continuum of care in maternal health services can reduce maternal and neonatal mortality by improving the health status of women and newborn babies [54].

This systematic review revealed that the magnitude of maternal mortality ratio among intervention group MMR = 323/100,000live births verses among controlled group MMR = 431/100,000live births. However, this finding is lower than MMR reported by EDHS 2016 (420 deaths per 100,000 live births) [39] and Global MMR documented by lancet 2006 (400 deaths per 100,000 live births) [55].

Similarly, this review identified that completion of continuum of care in maternal health services were not a statistically significant association with the reduction of maternal mortality. Even though, the interventions were not a statistically significant, those intervention packages are clinically decreasing the risk of maternal mortality by 26%. This evidence supported by evidence in Japan [48]. Moreover, in developing and low-middle income countries documented that 54% of maternal deaths avert per year [9]. Evidence in India revealed that effective implementation of continuum of care in maternal and child health programs is crucial to avert avoidable maternal and neonatal death [46]. Hence, the reduction of maternal and neonatal mortality is a good parameter for improvement of population health [56].

## 5 Limitations of the review

The limitations of this review were the absence of case – control and the sufficient amount of cohort and interventional study design did not obtain in the review; in some extent, the result of the review may have buried. The heterogeneity of the study articles showed statistical significant, but the source of heterogeneity did not identify. Thus, the interpretations of the finding need to be with caution.

This review focused on English language and published articles only. Because of the absence of other language and unpublished materials, the findings of this review may be under or over estimate.

Majority of studies retrieved from low-income countries and the searching engine did not retrieve the articles from developed countries, which blurred the generalization of the result for worldwide, particularly for developed and middle income countries.

Finally, the findings of this review should be interpreted in the circumstance of both inherent limitations of the original studies and the current systematic review and meta-analysis.

## 6 Conclusion

Based on the review results, we concluded that completion of a continuum of care in maternal health services may have a significant impact on the reduction of maternal and neonatal mortality. Hence, completion of continuum of care in maternal health services were significantly reduced neonatal mortality by 16% and clinically reduced maternal mortality by 26%. Therefore, this review recommends that interventional and follow-up studies will be encouraged to come up with more precise effects of completion of a continuum of care in maternal health services on maternal and neonatal mortality. However, the evidence found in this review was sufficient to recommend, so we recommend strengthening and effective implementation of continuum of care in maternal health services to improve maternal and neonatal health care outcomes.

## Author contribution statement

Muluwas Amentie Zelka: Conceived and designed the experiments; Performed the experiments; Analyzed and interpreted the data; Contributed reagents, materials, analysis tools or data; Wrote the paper.

Alemayehu Worku Yalew, Gurmesa Tura Debelew: Conceived and designed the experiments; Performed the experiments; Analyzed and interpreted the data; Contributed reagents, materials, analysis tools or data; Wrote the paper.

## Data availability statement

Data included in article/supp. material/referenced in article.

## Additional information

No additional information is available for this paper.

## Declaration of competing interest

The authors declare that they have no known competing financial interests or personal relationships that could have appeared to influence the work reported in this paper

## List of abbreviations

ANC: Antenatal Care
CI: Confidence Interval
COC: Continuum of Care
ICD – MM: International Classification of Diseases Maternal Mortality
ICD – PM: International Classification of Diseases Perinatal Mortality
LBs: Live Births
LMICs: Low-middle Income Countries
MMR: Maternal Mortality Ratio
NMR: Neonatal Mortality Rate
PICO: Participants, Interventions, control/comparison and Outcomes
PNC: Postnatal Care
PRISMA: Preferred Reporting Items for Systematic Reviews and Meta-analysis
RCT: Randomized Controlled Trials
RevMan: Review Manager Software
RR: Relative Risk
SBAs: Skilled Birth Attendants
SDGs: Sustainable Development Goals
